# Assistants' Manners and Nurses' Fees

**Published:** 1892-10-29

**Authors:** H. Rainsford


### Editor's Letter Box.

[Our correspondents are reminded that prolixity of statement is a great bar to publication, and that brevity of style and conciseness of statement greatly facilitate early insertion.]

### ASSISTANTS' MANNERS AND NURSES' FEES.

Sir,—Thanks for specimen copy of your paper. I have read with pleasure your leader on "The Practical Man." *Apropos* of that I once had a dispenser, a third year's student at St. Mary's, who informed me that in his opinion a senior student was a much better doctor than the ordinary general practitioner. Perhaps you will pardon my saying that I cannot see why there should be any articles on treatment in a weekly intended for nurses. Nurses are much too ready as it is to interfere in the treatment of cases without being coached up by you; their duty is to carry out all instructions given them by the medical attendant, and not to go in for treating the cases themselves. In another part of the paper you allude to some persons who object to paying two guineas per week for a nurse; far be it from me to say that a good nurse is not worth that or any sum the patient can afford, but in Dublin the best of trained nurses never ask for more than one guinea per week, and a very skilful sister of the Red Cross Association (I think situated in Harcourt Street) attended my father through a severe attack of pneumonia for that sum. It seems strange there should be such a diversity in the cost in two places so close as London and Dublin.—Yours, &c.,

